# From fear to innovation: the mediating roles of curiosity and grit in the relationship between fear of academic failure and creative problem-solving among Chinese undergraduates

**DOI:** 10.3389/fpsyg.2025.1625134

**Published:** 2026-01-09

**Authors:** Lanfeng Zhou, Liying Xie, Xinyu Wang

**Affiliations:** 1 College of Marxism, Putian University, Putian, Fujian, China; 2 Party School and Administrative School of CPC, Putian, Fujian, China; 3 School of Education and Psychology, Minnan Normal University, Zhangzhou, Fujian, China

**Keywords:** China, creative problem-solving, curiosity, fear of academic failure, grit, undergraduates

## Abstract

**Background:**

Fear of Academic Failure (FoAF) is a common emotional experience among undergraduates and is often associated with differences in academic performance and problem-solving. While the relationship between fear and academic outcomes is well-documented, less is known about how FoAF relates to creative problem-solving (CPS). This study explores the roles of curiosity and grit as mediators in this relationship.

**Methods:**

A cross-sectional, correlational study was employed with a sample of undergraduate students from three universities in Putian City, China. Participants completed self-report measures assessing FoAF, Curiosity, Grit, and CPS. Data were analyzed using structural equation modeling to test the direct and indirect associations of FoAF on CPS via curiosity and grit.

**Findings:**

The results revealed that FoAF was modestly positively associated with CPS (β = 0.19, 95% CI [0.091, 0.274]). Curiosity (β = 0.118) and grit (β = 0.176) also showed positive associations with CPS, and both mediators showed significant indirect associations. The pattern of coefficients indicates that the FoAF–CPS association via grit was comparatively stronger than via curiosity.

**Conclusion:**

This study offers a deeper understanding of the psychological mechanisms underlying CPS among undergraduate students. While curiosity sparks initial engagement, grit underpins the ability to solve problems creatively. These findings suggest that cultivating grit in students, particularly those facing academic challenges, may be more effective than focusing solely on curiosity. By emphasizing grit alongside curiosity, educational interventions can better support students in navigating academic obstacles and developing the problem-solving skills necessary for success in their studies and beyond.

## Introduction

Problem-solving is an essential cognitive skill that underpins academic success, particularly in higher education (Saxena et al., 2014). As students navigate complex tasks, they must apply Creative Problem-Solving (CPS) strategies that involve cognitive flexibility and effective decision-making (Balázs, 2019). Students frequently encounter challenges that test their problem-solving abilities in academic environments, ranging from routine academic tasks to more complex assignments (Balázs, 2019). The ability to solve problems creatively has been identified as a key predictor of academic success, allowing students to tackle challenges with novel solutions and sustain engagement in the face of academic stress (Pullu and Gömleksiz, 2021). Among the myriad challenges university students face, Fear of Academic Failure (FoAF) is one of the most prominent emotional experiences that can significantly impact their academic performance and cognitive functioning (Jackson, 2017). FoAF, often driven by academic pressures (Lewis, 2023), is particularly acute in undergraduate students, who face the added stress of transitioning to a new, more demanding academic environment. This fear can manifest in several ways, including avoidance of tasks (Bartels and Magun-Jackson, 2009), procrastination (Schouwenburg, 1992), or diminished motivation to engage in academic work (Nakhla, 2019). These responses can severely undermine students' ability to engage with learning tasks, particularly those requiring CPS skills. While the negative consequences of fear of failure are well-documented, recent research has also suggested that it may serve as a motivational factor, propelling students to invest greater effort in their academic work. Fear of failure can thus paradoxically foster greater engagement and persistence in problem-solving tasks (Gao et al., 2024). However, the relationship between fear of failure and problem-solving abilities remains complex, with positive and negative outcomes depending on individual coping mechanisms and emotional regulation. This dynamic presents an opportunity to explore how FoAF relates to CPS through psychological traits such as curiosity and grit, which are critical for academic perseverance.

Undergraduate students are particularly susceptible to FoAF during the transition from secondary education to university, when academic expectations are significantly heightened. This stage in their educational journey often involves increased academic stress, heightened performance expectations, and a greater focus on personal achievement, all of which contribute to a higher incidence of academic fear (Alkhazaleh and Mahasneh, 2016). These factors make undergraduates an ideal population for studying the relationship between FoAF and problem-solving. Moreover, undergraduate students are at a critical developmental stage, learning academic content and essential cognitive and emotional skills, such as curiosity and grit, which are integral to CPS. Given the pivotal role of problem-solving in academic success, understanding the mechanisms by which FoAF relates to CPS could provide valuable insights to enhance learning outcomes and foster resilience among students facing academic challenges.

## Linking FoAF to CPS

In academic contexts, problem-solving abilities are crucial for student success, especially when navigating complex or high-stakes challenges (Stadler et al., 2018). One emotional factor that may interact these abilities is the FoAF, a common experience among undergraduates. FoAF can affect students' willingness to engage in demanding academic tasks, potentially shaping their motivation, creativity, and problem-solving behavior. Although fear is often viewed as a hindrance, it may also act as a motivating force, pushing students to exert more effort to avoid failure-related outcomes (Henry et al., 2019). The present study examines whether FoAF directly predicts CPS among undergraduates, based on the premise that fear may trigger compensatory engagement strategies to manage anticipated academic threats (Tan et al., 2019). This hypothesized relationship is informed by Self-Regulation Theory, which posits that emotionally activated students, such as those experiencing FoAF, mobilize goal-directed regulation strategies to manage academic demands (Zimmerman, 2002; Wagner and Heatherton, 2015). In this framework, FoAF may encourage students to persist and adopt adaptive coping behaviors, laying the groundwork for grit as a persistence-oriented mediator. Similarly, Self-Determination Theory (SDT) suggests that when students experience FoAF but retain a sense of competence and autonomy, fear can be internalized as a motivational force that stimulates curiosity and exploration. Together, these theories explain why FoAF may not simply hinder performance but instead channel students toward curiosity-driven engagement and grit-driven perseverance in CPS (Ryan and Deci, 2000).

Empirical studies have similarly noted the complex role of fear in academic settings. For example, Elliot and McGregor (2001) found that FoAF is often associated with performance-avoidance goals, which may lead to disengagement. However, other studies have shown adaptive effects. Nakhla (2019) reported that fear of failure can lead to heightened academic focus and problem-solving when students perceive the fear as a challenge rather than a threat. In addition, Duru et al. (2024) found that emotion regulation difficulties mediate the relationship between FoAF and academic outcomes, such as procrastination and satisfaction, underscoring how self-regulatory capacity can determine whether fear becomes disabling or motivating. Complementing this, a recent meta-analysis found that effective emotion regulation strategies enhance learning, reduce burnout, and promote academic resilience, suggesting that self-regulation has a protective and performance-enhancing function in high-stress educational contexts (Iuga and David, 2024). These findings indicate that FoAF may not universally hinder performance; its effects depend on contextual and individual variables. The current study hypothesizes that FoAF may be positively associated with CPS. While fear is often perceived as maladaptive in academic contexts, it may enhance students' engagement and encourage creative responses to academic stressors under certain motivational conditions. This dual role of fear—both a barrier and a motivator—has been highlighted in emerging research but remains underexplored in the literature on creativity and academic performance.

## Mediating role of curiosity

Curiosity has long been recognized as a key psychological trait that motivates individuals to seek novel information and engage with unfamiliar challenges (Kidd and Hayden, 2015). Curiosity supports adaptive learning behaviors in academic settings by encouraging students to explore alternative perspectives and persist in problem-solving tasks (Peterson, 2020; Markey and Loewenstein, 2014). Defined as a desire to reduce uncertainty and expand knowledge, curiosity is often aligned with intrinsic motivation (Rinkevich, 2014). This study posits that curiosity may mediate the relationship between FoAF and CPS. While FoAF is typically associated with academic stress, it may co-occur with increased curiosity, particularly when students seek new strategies to reduce uncertainty and regain control over academic outcomes. From the perspective of SDT, FoAF may heighten curiosity when students perceive academic challenges as opportunities to demonstrate competence. In this way, curiosity operates as an intrinsic motivational response that translates into deeper engagement and flexible problem-solving (Deci and Ryan, 2000). In stressful academic environments, some students may experience FoAF alongside a drive to master difficult content, especially when they feel capable of overcoming challenges. In such contexts, curiosity may function as an adaptive response to academic tension. Prior research supports this interpretation. For instance, Pluck and Johnson (2011) demonstrated that curiosity facilitates exploration and engagement, particularly when students face novel or challenging material. Other studies have shown that emotional discomfort, including anxiety or fear, can motivate students to seek novel information to reduce uncertainty (Tariq, 2024; Singh and Manjaly, 2022). Gross et al. (2020) emphasized that curiosity plays a central role in CPS by encouraging flexible thinking and exploration of new solutions. In this way, curiosity may not arise directly from fear, but may emerge as part of a broader motivational process through which students regulate their emotional responses to academic demands. Finally, while curiosity may help initiate engagement, it may not be sufficient to sustain prolonged academic effort. In the presence of FoAF, curiosity may need to be coupled with persistence-oriented traits, such as grit, to fully support CPS. Thus, curiosity is positioned in this model as a possible bridge between fear and creative engagement—one that enables exploration but may rely on other traits to translate intention into sustained action.

## Mediating role of grit

Grit, defined as perseverance and passion for long-term goals, is a critical trait influencing students' academic success and problem-solving (Li and Li, 2021). In higher education, where undergraduates frequently face FoAF, grit enables them to maintain focus and resilience in the face of challenges, thereby supporting CPS (Hoidn and Kärkkäinen, 2014). Unlike curiosity, which initiates engagement, grit sustains effort over prolonged tasks, making it central to how students confront and overcome FoAF (Wang, 2021; Allen et al., 2021; Birr et al., 2024). The concept of grit has gained significant attention in recent decades, with Duckworth et al. (2007) pioneering its exploration in the academic domain. They defined grit as a trait combining passion for long-term goals with the perseverance required to achieve them. Their research demonstrated that individuals with higher levels of grit are more likely to succeed in long-term, effortful tasks, even when faced with significant obstacles. In academic settings, grit is particularly important for students, as it encourages them to maintain their focus and effort over time, even in the face of academic challenges such as FoAF (Lee, 2017). Research has shown that students with higher grit levels tend to perform better in problem-solving tasks, particularly in high-pressure situations (Schmidt et al., 2018). Li and Li (2021) emphasized that grit is crucial for overcoming academic setbacks, as it enables students to persevere and remain engaged in the problem-solving process, even when initial efforts do not yield immediate success. This finding is particularly relevant for undergraduate students, who often face the transition from high school to university and the increased academic demands that accompany it. The FoAF that many students experience during this transition may amplify the need for grit as a buffer that helps students persist in the face of academic stress. The mediating role of grit in the FoAF–CPS relationship can be explained by two complementary frameworks. Self-Regulation Theory emphasizes that learners who face academic stress can regulate emotions and maintain goal-directed effort; grit embodies this capacity by enabling students to persist despite fear or setbacks (Zimmerman, 2002). In parallel, Achievement Goal Theory highlights that gritty students are more likely to adopt mastery-oriented goals, reframing fear as a challenge to overcome rather than a threat to avoid (Ames, 1992). Together, these perspectives suggest that grit functions as a self-regulatory mechanism and motivational orientation that transforms FoAF into sustained engagement, facilitating creative problem-solving.

### This study

Despite the growing interest in FoAF, there is a limited understanding of how this fear relates to underlying psychological traits in connection with CPS in undergraduates. Existing literature has primarily focused on the direct association between fear and academic performance, neglecting the roles of curiosity and grit as potential mediators in this relationship. The dynamic processes by which FoAF shapes students' problem-solving behaviors remain underexplored, particularly how curiosity, often linked to exploration and learning, and grit, associated with perseverance, enhance academic resilience and problem-solving. While previous studies have separately examined the roles of curiosity and grit in educational success, their combined impact on CPS responses to fear has not been adequately addressed. This study aims to bridge this gap by examining how FoAF is associated with CPS, with curiosity and grit as mediating factors. By exploring these interactions, the study will shed light on how motivational traits are associated with academic problem-solving beyond cognitive skills, offering valuable insights for developing interventions that foster resilience, creativity, and perseverance in students under academic stress.

## Methodology

### Study design

This study employed a cross-sectional design to explore the relationship between various psychological factors and academic outcomes among undergraduate students. Data collection occurred between January and February 2025, and the survey was conducted using WeChat, a widely used online platform that facilitates the participation of university students in Putian City, China.

### Participants

Undergraduate students from three universities in Putian City, China, participated in the study. The universities were selected based on administrative consent from each institution before the study's commencement. The total sample consisted of students from various academic disciplines, ensuring a representative cross-section of undergraduate populations and minimizing selection bias. This study excluded international students because they were not part of the sample, given potential differences in academic experiences and cultural backgrounds that could have influenced the study outcomes.

### Sampling methodology

A convenience sampling method was employed to recruit participants for the study. This method was chosen due to its feasibility and the practical constraints of recruiting from multiple institutions. The universities provided administrative consent to distribute the survey to their students via WeChat, an online platform that Chinese students use. Participation was voluntary, and the survey invitation was sent to all undergraduate students enrolled at the participating universities.

While convenience sampling does not guarantee random selection, it was the most appropriate method given the study's logistical constraints. To increase the sample's diversity across disciplines, students from various academic programs and departments were invited to participate. This approach helps to ensure a representative cross-section of undergraduate students, encompassing a wide range of academic disciplines and reducing bias toward any particular field of study.

### Eligibility criteria

Participants in this study had to meet specific inclusion criteria to ensure the sample was representative of the target population. Only undergraduate students enrolled in one of the three participating universities in Putian City, China, were eligible to participate. Additionally, participants had to be native Chinese speakers, as the survey was administered in Chinese to ensure that all questions were clearly understood. The study also included exclusion criteria to minimize confounding factors. International students were excluded from the study due to potential differences in their academic experiences and cultural backgrounds, which could have influenced outcomes in ways that were not representative of the domestic student population. Furthermore, students who self-reported having a mental disability or those currently taking mental health medication were excluded from the study. This exclusion was implemented to avoid any bias or confounding effects that might arise from mental health conditions or the impact of drugs on cognitive or emotional responses, which could distort the study's results. These eligibility criteria were carefully designed to ensure the study focused on healthy undergraduate students with relevant academic and psychological experiences, enabling a clear and valid examination of the factors under study.

### Data collection

Data were collected using a self-administered online questionnaire designed to capture a range of variables related to academic engagement, psychological factors, and problem-solving abilities. WeChat enabled participants to complete the survey at their convenience, thereby increasing the likelihood of obtaining a larger, more diverse sample. The questionnaire was available in Chinese to accommodate the linguistic preferences of the student population, thereby reducing potential language barriers that could affect the accuracy and validity of the responses.

### Sample size determination

The sample size for this study was determined based on the desired statistical power and the expected effect size. A power analysis was conducted to ensure the sample size was sufficient to detect meaningful effects. Using Cohen's guidelines for power analysis (Cohen, 1992), a power level of 0.80 was targeted, with an alpha level of 0.05. Based on these parameters and considering the expected medium effect size (Cohen's *d* = 0.5), a minimum sample size of 350 participants was required for reliable analysis. The survey link was distributed to approximately 1,654 undergraduate students across the three universities. Of these, 1,242 students began the survey, and 1,129 provided valid, complete responses, resulting in an approximate response rate of 68.26%. The final sample size of 1,129 valid questionnaires far exceeds this minimum requirement, ensuring sufficient statistical power for a mediation analysis, including regression and correlation tests. This large sample also increases the generalizability of the results across the diverse undergraduate student population in the three participating universities. Thus, the study was adequately powered to detect relationships between the variables of interest, ensuring that the findings are statistically significant and not due to random chance.

### Data cleaning and screening

After data collection, all responses were cleaned and screened to ensure the validity and integrity of the dataset. Of the original responses, 1,129 valid questionnaires were retained after eliminating incomplete or invalid submissions. A small proportion of less than 10% of the data was missing, primarily due to skipped questions. To handle this missing data, multiple imputation techniques were used to fill in missing responses. This method helps ensure that the dataset remains robust and minimizes bias in the analysis, adhering to best practices for missing data treatment in cross-sectional studies.

### Ethical considerations

This study was conducted following the ethical principles of the Declaration of Helsinki. Before data collection, ethical approval was obtained from the Research Ethics Committee of Putian University (No. 6133/R). Informed consent was obtained electronically. Participants were informed about the voluntary nature of their involvement, assured of their anonymity and confidentiality, and given the right to withdraw from the study at any point without penalty.

## Measuring instruments

### Sociodemographic

Sociodemographic information, including age, gender, year of study, and academic discipline, was collected to describe the study sample. The final sample (*N* = 1,129) comprised undergraduate students aged 18–24 years, with a mean age of approximately 20.3 years (SD = 1.46). Of the participants, 612 (54.2%) identified as female and 517 (45.8%) as male. Regarding academic year, 28.1% were first-year students, 26.7% were second-year students, 23.5% were third-year students, and 21.7% were fourth-year students. Participants were enrolled across a wide range of academic disciplines: Social Sciences (*n* = 376; 33.3%), Engineering and Technology (*n* = 294; 26.0%), Business and Economics (*n* = 189; 16.7%), Education and Psychology (*n* = 142; 12.6%), and Arts and Humanities (*n* = 128; 11.3%).

### Fear of academic failure

The Chinese version of the Fear of Failure in Learning Scale (FFLS) is designed to assess university students' fear of academic failure (Chen et al., 2024). This scale assesses cognitive and emotional responses to educational failure, including anxiety, worry about performance, and insecurity about academic abilities. The FFLS consists of 26 items rated on a 5-point Likert scale, ranging from 1 (strongly disagree) to 5 (strongly agree). Higher scores on the FFLS indicate greater levels of FoAF, which can negatively affect motivation and educational engagement and lead to behaviors such as procrastination. The scale has strong internal consistency and is widely used among Chinese university students (Chen et al., 2024; Choi, 2025).

### Curiosity

The Curiosity and Exploration Inventory–II (CEI-II), developed by Kashdan et al. (2004), is a psychometric tool designed to assess two dimensions of curiosity: epistemic curiosity (the desire to acquire knowledge) and diverse curiosity (the tendency to seek new experiences). It consists of 10 items, with responses rated on a 5-point Likert scale. The Chinese version of the CEI-II has been validated for use with Chinese university students in Hong Kong (Ye et al., 2015), demonstrating strong reliability (Cronbach's alpha = 0.85) and factorial validity, confirming the two-factor structure. The scale has been shown to predict academic engagement and openness to new learning experiences in university contexts. This validated version is ideal for measuring curiosity in Chinese undergraduates, providing a reliable tool for research on academic curiosity and engagement.

### Grit

Grit was assessed using the Original Grit Scale (Grit-O), developed by Duckworth et al. (2007), which measures two key facets: perseverance of effort and consistency of interest. For the present study, we adopted the Chinese-translated version validated by Hou et al. (2024), adapted explicitly for Chinese young adults in higher education. This version has demonstrated robust psychometric properties, including construct validity, internal consistency, measurement invariance across time, and predictive validity in university student populations across various disciplines. The Chinese Grit-O consists of 12 items rated on a 5-point Likert scale ranging from 1 (Not at all like me) to 5 (Very much like me). Items were divided evenly between the two subscales: Perseverance of Effort (e.g., “I am a hard worker”) and Consistency of Interest (e.g., “New ideas and projects sometimes distract me from previous ones”—reverse scored). The reported Cronbach's alpha coefficients in prior research ranged from 0.76 to 0.83 across subscales, and the scale showed good model fit in confirmatory factor analysis (CFA) among undergraduate samples (Wu et al., 2022; Zhang et al., 2022; Chen et al., 2018). This adaptation ensures cultural and linguistic appropriateness for Chinese university students and has been endorsed as a reliable and valid tool in related studies within Mainland China.

### Creative problem-solving

The Problem-Solving Inventory (PSI), developed by Heppner and Petersen (1982), is a widely used tool to assess an individual's problem-solving style and confidence in solving personal and academic challenges. The inventory evaluates the cognitive and behavioral aspects of problem-solving, measuring an individual's self-reported problem-solving confidence and approach-avoidance tendencies. For the present study, the Chinese version of the PSI, validated by Chan (2001), was used. The scale consists of two dimensions: Problem-Solving Confidence—the belief in one's ability to solve problems successfully. Approach-Avoidance Style—the tendency to actively engage with problems (approach) or avoid confronting them (avoidance), with 35 items, rated on a 6-point Likert scale, with higher scores indicating greater confidence and a more proactive approach to problem-solving. This version of the PSI has shown strong internal consistency and good construct validity, making it a reliable measure of problem-solving ability and psychological adjustment among Chinese students. This instrument has been successfully applied in a variety of educational contexts (Sahin et al., 1993; Heppner and Anderson, 1985; Chaji et al., 2022).

### Statistical analysis

All statistical analyses were conducted using SPSS (v28) and AMOS (v29). Descriptive statistics and Pearson's correlation coefficients were first computed to examine the basic relationships among key variables. Age and gender were included as covariates across analyses. Structural Equation Modeling (SEM) was employed using AMOS to test the hypothesized mediation model. SEM was selected as the primary analytic approach due to its ability to simultaneously test complex relationships among multiple latent constructs. The analysis followed a two-step approach: First, Confirmatory Factor Analysis (CFA) was conducted to evaluate the measurement model, ensuring the reliability and validity of the latent constructs. Model fit was assessed using multiple indices, including the χ^2^/d*f* ratio, CFI, TLI, RMSEA, and SRMR. Second, the structural model was tested to evaluate direct and indirect associations among the study variables. Bootstrapping with 5,000 resamples was used to estimate 95% confidence intervals (CI) for all direct and indirect paths, providing robust significance testing of the mediation paths. A parallel mediation framework was specified, allowing curiosity and grit to function as simultaneous mediators in the relationship between FoAF and CPS. All estimates were reported as standardized path coefficients (β), and statistical significance was determined using *p*-values and bootstrapped confidence intervals.

## Results

### Testing for common method bias

To address the potential issue of common method bias, we conducted Harman's Single Factor Test. An exploratory factor analysis (EFA) was performed on all of the study's constructs. If a single factor had emerged and accounted for most of the variance (typically greater than 40%), it would suggest the presence of common method bias. However, the results showed that multiple factors emerged, and no single factor dominated the variance, indicating that common method bias was not a significant concern in our data.

### Curvilinear sensitivity check

To assess potential nonlinearity in the FoAF–CPS association, CPS was compared across quartiles of FoAF, and polynomial contrasts were tested; a regression including a centered quadratic FoAF term was also fit. CPS differed across FoAF quartiles, *F*_(3, 618)_ = 5.56, *p* = 0.006, $n_{p}^{2}$ = 0.020. The linear contrast was significant, *F* = 11.35, *p* = 0.001, whereas the quadratic and cubic contrasts were not, *F* = 0.74, *p* = 0.391 and *F* = 0.09, *p* = 0.78, respectively. In a regression including a centered quadratic FoAF term, the quadratic coefficient was non-significant, *b*_2_ = −0.05, *SE* = 0.048, *p* = 0.41, with negligible improvement in fit (Δ*R*^2^ = 0.002). These checks indicated that the FoAF—CPS association was approximately linear over the observed range.

### Preliminary analysis

Table 1 demonstrates the four constructs' discriminant validity, reliability, and construct validity analysis results. The basement and ceiling effects were minimal across all constructs, with values well below the 15% threshold, indicating sufficient response variability. Reliability was strong, with McDonald's omega ranging from 0.83 to 0.88 and Cronbach's alpha values between 0.85 and 0.89, surpassing the 0.7 cutoff for good internal consistency. Ferguson's delta values also exceeded 0.9, supporting strong discriminant validity between the constructs.

**Table 1:** Discriminant validity, reliability, and construct validity analysis results.

	**FoAF**	**Curiosity**	**Grit**	**CPS**	**Cut off**
Basement effect	2.7%	2.5%	2.8%	3.5%	< 15%
Ceiling effect	3.4%	2.5%	3.1%	3.9%	< 15%
McDonald's omega	0.88	0.86	0.83	0.88	≥0.7
Cronbach's alpha	0.89	0.88	0.85	0.89	≥0.7
Ferguson's delta	0.91	0.90	0.89	0.92	≥0.9
CFI	0.96	0.94	0.91	0.94	≥0.95
TLI	0.95	0.94	0.92	0.94	≥0.95
RMSEA	0.038	0.032	0.035	0.039	< 0.08
SRMR	0.041	0.029	0.034	0.041	< 0.08
χ^2^/d*f*	1.32	1.65	1.18	1.46	< 3
AVE	0.61	0.62	0.64	0.63	>0.5

d*f*, degrees of freedom; CFI, comparative fit index; TLI, Tucker-Lewis index; RMSEA, root mean square error of approximation; SRMR, standardized root mean squared residual; AVE, average variance extracted; FoAF, fear of academic failure; SC, creative problem-solving.

**Figure 1:**
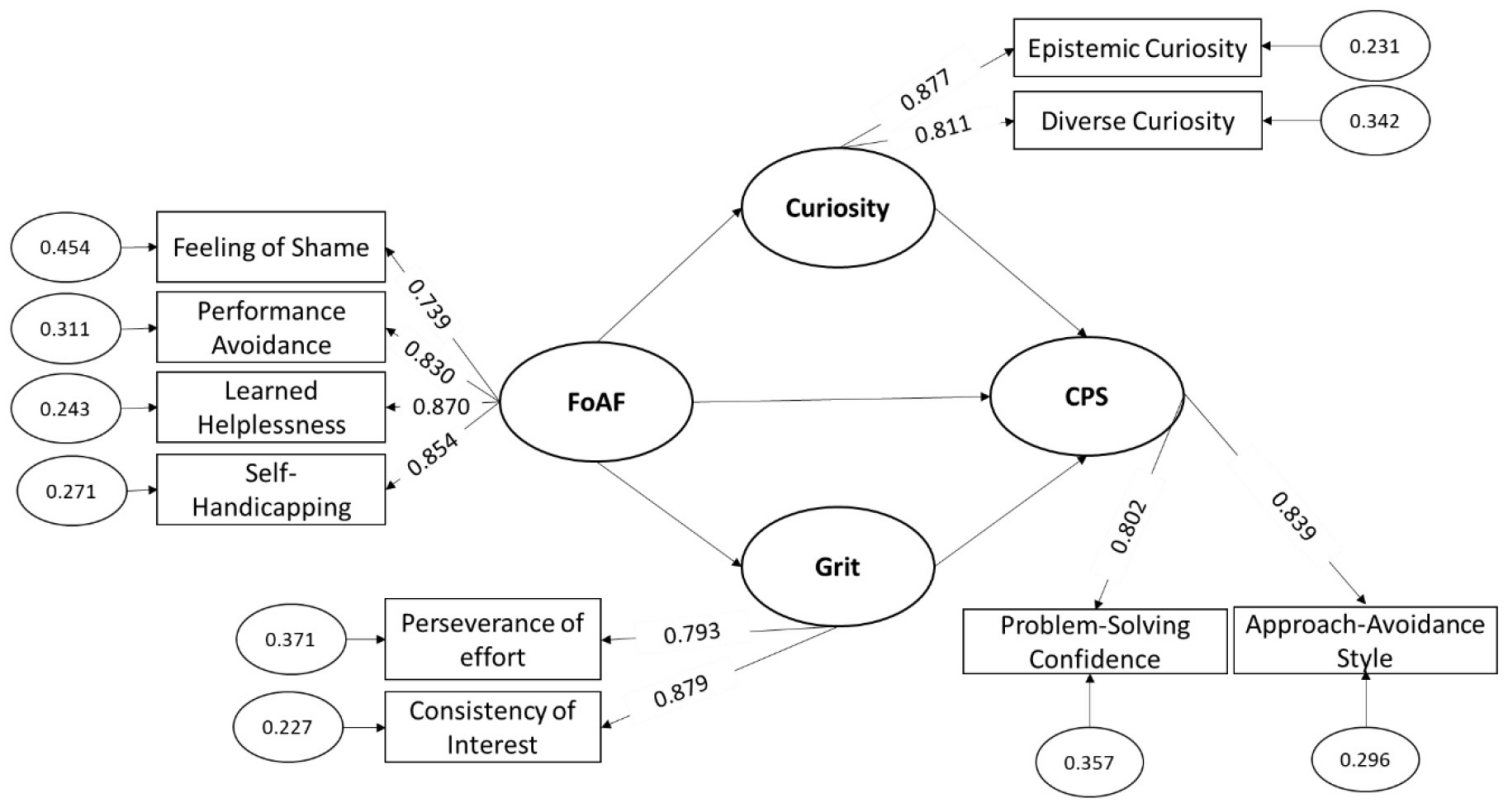
Structural equation modeling of the hypothesized model with standardized coefficients.

In terms of construct validity, all model fit indices were excellent. CFI values ranged from 0.91 to 0.96, TLI values from 0.92 to 0.95, and RMSEA values below 0.08, indicating a good fit. Finally, SRMR values were below the 0.08 threshold for all constructs, further supporting the model's validity. Overall, the scales used in the study were reliable, valid, and fit the data well. In addition, the measurement model demonstrated satisfactory factor loadings for all observed indicators (ranging from 0.739 to 0.879), with corresponding residual variances within acceptable limits, supporting good construct validity for the latent variables (Figure 1).

### Pearson's bivariate correlation analysis

Table 2 presents the descriptive statistics and bivariate correlations for the four constructs: FoAF, curiosity, grit, and CPS. Mean scores for all variables indicated moderate levels across the sample. Pearson correlations revealed that FoAF was significantly positively correlated with curiosity (*r* = 0.472, *p* < 0.001) and grit (*r* = 0.511, *p* < 0.001), suggesting a moderate association among these variables. While this positive correlation may reflect a potential motivational role of FoAF, the direction and nature of the relationship cannot be determined from correlational data alone. It is also important to note that fear can facilitate and inhibit effects, as suggested in prior literature (Pekrun, 2006). Additionally, curiosity and grit were moderately correlated (*r* = 0.291, *p* < 0.01), indicating that these constructs share some common variance. However, this relationship should not be interpreted as one trait enhancing or predicting the other. Regarding CPS, FoAF was weakly correlated (*r* = 0.253, *p* < 0.01), whereas curiosity (*r* = 0.463, *p* < 0.001) and grit (*r* = 0.569, *p* < 0.001) showed stronger positive correlations, suggesting that students who reported higher levels of curiosity and grit also tended to report stronger CPS abilities.

**Table 2:** Descriptive and bivariate correlational analysis.

	***M* ±SD**	**FoAF**	**Curiosity**	**Grit**	**CPS**
FoAF	3.53 ± 0.71	1			
Curiosity	3.22 ± 0.85	0.472^***^	1		
Grit	3.25 ± 0.65	0.511^***^	0.291^**^	1	
CPS	3.17 ± 0.76	0.253^**^	0.463^***^	0.569^***^	1

^***^p < 0.001, ^**^p < 0.01. FoAF, fear of academic failure; SC, creative problem-solving.

### Direct path analysis

Table 3 presents the direct path analysis, with age and gender included as covariates. The results showed the standardized path coefficients (β) and *p*-values to determine each path's statistical significance. The direct association of FoAF on CPS was β = 0.19, with a *p*-value < 0.05, indicating a statistically significant, albeit moderate, positive association. This indicates that FoAF was directly and positively associated with CPS abilities, although the association is relatively small. On the other hand, the path from FoAF to curiosity showed a more substantial association (β = 0.32, *p* < 0.001), indicating a significant positive relationship, such that higher FoAF was associated with higher curiosity, a pattern consistent with students experiencing fear may seek new ways to approach problems or learn more, possibly as a way of overcoming academic challenges.

**Table 3:** Direct path analysis (age and gender as covariates).

**Direct path**	**Standardized estimate β**	***p*-value**	**Bootstrapped 95% CI**	**Decision**
FoAF → CPS	0.19	< 0.05	[0.091–0.274]	Supported
FoAF → Curiosity	0.32	< 0.001	[0.214–0.483]	Supported
FoAF → Grit	0.41	< 0.001	[0.322–0.542]	Supported
Curiosity → CPS	0.37	< 0.001	[0.257–0.493]	Supported
Grit → CPS	0.43	< 0.001	[0.364–0.604]	Supported

In addition, the direct association of FoAF on Grit (β = 0.41, *p* < 0.001) was the strongest among the direct paths, indicating a strong positive relationship. This suggests that students who reported higher FoAF also reported higher grit (perseverance) to push through academic difficulties. Curiosity had a significant positive association with CPS (β = 0.37, *p* < 0.001), indicating that higher curiosity was associated with higher CPS scores. Similarly, Grit also showed a strong positive association with CPS (β = 0.43, *p* < 0.001), indicating that grittier students, who persist in their efforts, are more likely to demonstrate enhanced problem-solving abilities. To strengthen the robustness of the results, bootstrapped 95% confidence intervals (CI) with 5,000 resamples were calculated for all direct paths in the model. As shown in Table 3, all confidence intervals excluded zero, indicating statistically significant associations across all direct paths.

### Parallel mediation analysis

**Figure 2:**
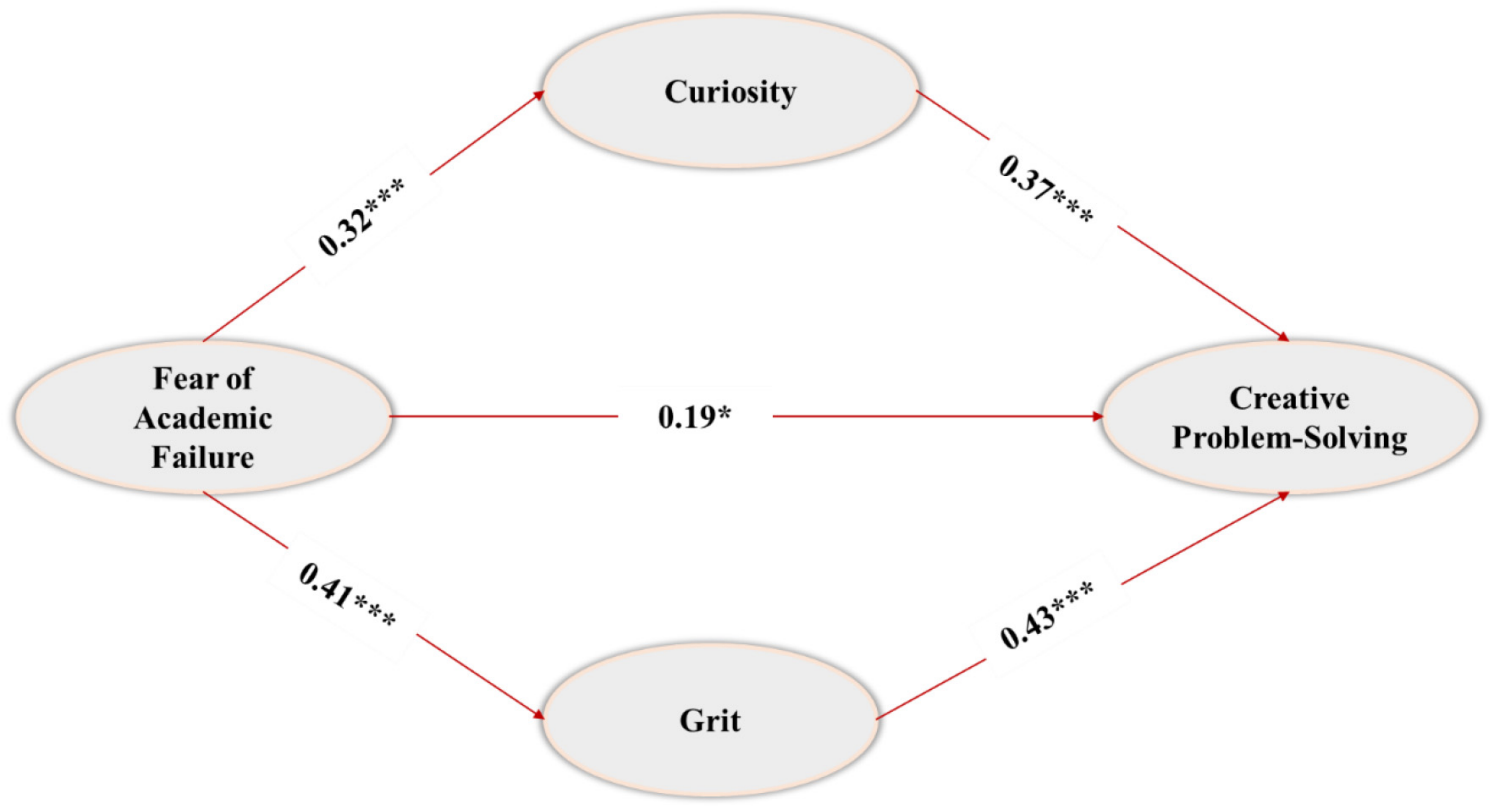
Standardized path coefficients and significance of the parallel mediation model (*p* < 0.001, *p* < 0.05).

Table 4 presents the indirect path analysis, with age and gender included as covariates (Figure 2). The indirect association of FoAF → Curiosity → CPS was β = 0.118, with a 95% CI of [0.101, 0.254], indicating statistical significance as the interval excluded zero. This supports a significant indirect association between FoAF and CPS through Curiosity. Likewise, the indirect association of FoAF → Grit → CPS was β = 0.176, 95% CI [0.144, 0.285], indicating a significant indirect pathway. These findings suggested that curiosity and grit are statistically significant mediators in the relationship between FoAF and CPS. While the indirect association through grit was somewhat more substantial, the data do not allow for causal conclusions or insights into the underlying psychological processes. Instead, the results reflect co-occurrence patterns among self-reported fear of failure, curiosity, grit, and CPS within the sample. The *R*^2^ value of 0.36 and *F* statistic of 309.11 (*p* < 0.001) indicated that the model accounts for 36% of the variance in CPS. Additionally, the direct association from FoAF to CPS remained significant in the full model (β = 0.19, *p* < 0.05, 95% CI [0.091, 0.274]), supporting the interpretation that both direct and indirect paths contribute to the relationship between FoAF and CPS.

**Table 4:** Indirect path analysis (age and gender as covariates).

**Indirect path**	**Standardized estimate β**	**Bootstrapped 95% CI**	**Decision**
FoAF → Curiosity → CPS	0.118	[0.101–0.254]	Partial mediation
FoAF → Grit → CPS	0.176	[0.144–0.285]	Partial mediation
*R^2^* = 0.36, *F* = 309.11^***^			

^***^p < 0.001.

## Discussion

The primary objective of this study was to examine how FoAF relates to CPS and whether this relationship is mediated by curiosity and grit among undergraduate students. The results indicated that curiosity and grit were statistically significant mediators in the relationship between FoAF and CPS. Notably, the indirect path through grit was somewhat more substantial than curiosity. FoAF was positively associated with curiosity and grit, and both traits were positively associated with CPS. These findings suggested that, within this model, grit accounted for a larger portion of the indirect association between FoAF and CPS than curiosity.

### Direct association of FoAF on CPS

A substantial body of research indicates that fear of failure is typically associated with lower creative performance under strong evaluative pressure and low control (Byron et al., 2010; Huang and Yu, 2025). Proposed explanations emphasize risk aversion, favoring safe, well-learned responses over exploration; fear-based perfectionism centered on concerns over mistakes that coincides with delay and avoidance; anxiety-linked fixed-ability beliefs that discourage persistence after setbacks; difficulty sustaining task-absorbed states; and a narrowed attentional focus oriented toward error prevention rather than idea generation (Amabile, 1983; Daker et al., 2023; Doyle, 2017; Goulet-Pelletier et al., 2022). Within this context, estimates in the present study are interpreted as correlational and context-bound rather than as evidence that fear improves creativity. The first hypothesis posited a direct association between FoAF and CPS. A significant but modest positive association was observed, implying that higher FoAF scores coincided with slightly higher CPS scores. Given the small standardized coefficient, this association is interpreted cautiously. One correlational explanation is that, in some academic settings, fear-related appraisals co-occur with task focus and persistence when students also report motivational engagement and self-regulatory effort. Prior evidence aligns with this possibility. Henry et al. (2019) demonstrated that higher fear of failure was associated with greater cognitive effort and more frequent problem-solving behaviors; Hong et al. (2019) found that students with higher emotional regulation, despite FoAF, reported stronger creative self-efficacy and motivation to resolve complex tasks; Wang and Jiang (2022) argued that emotional modulation enables students to channel fear into productive academic engagement; and Orakci (2023) reported that FoAF alone has a significant role in predicting CPS, shaped by academic motivation and self-efficacy. These findings complement our study, indicating that while FoAF is associated with problem-solving, it likely operates within a broader network of emotional and cognitive factors. Collectively, these studies suggest that FoAF can relate to problem-solving under certain conditions, particularly when moderated by self-belief or emotional strategies. The results of the current study are consistent with this broader literature, but they do not demonstrate that fear directly causes increased creativity or engagement. Instead, they suggest that, although modestly, FoAF is positively associated with CPS performance among students. This finding suggests that FoAF is only one of several emotional or cognitive variables associated with CPS outcomes.

From a theoretical perspective, the positive relationship between FoAF and CPS may be interpreted through the lens of self-determination theory and self-regulation theory. According to Ryan and Deci (2000) SDT, fear-induced stress can sometimes activate intrinsic motivation, particularly when students are internally driven to resolve uncertainty and affirm their competence. In academic contexts, this motivation may translate into greater persistence and engagement with problem-solving tasks. Similarly, self-regulation theory emphasizes how students manage their emotions, behaviors, and cognitive strategies to achieve academic goals. Within this framework, FoAF may prompt students to exert greater cognitive effort or adopt adaptive coping strategies—such as curiosity and grit—that support creative problem-solving. However, the relatively weak direct association of FoAF on CPS observed in this study suggests that while fear may catalyze academic engagement in some students, it is likely not the sole contributor. Other factors, including intrinsic traits such as curiosity or prior academic success, may play a more substantial role in shaping CPS among undergraduates.

### Indirect association of FOAF on CPS through curiosity

The results supported the second hypothesis, as the indirect path from FoAF to CPS via curiosity was statistically significant. This suggested that curiosity plays a mediating role in the relationship between FoAF and CPS among undergraduate students. The positive association between FoAF and curiosity indicated that students experiencing higher levels of FoAF tend to become more curious, which, in turn, enhances their problem-solving abilities. The positive link between FoAF and curiosity is well-supported by existing literature. Ryan and Deci (2000) argued that when individuals experience fear or anxiety, they often turn to intrinsic motivation as a way of coping, which in turn stimulates curiosity. Similarly, De Castella et al. (2013) found that students with high FoAF may use fear as a motivational driver to remain engaged and avoid failure. Henry et al. (2021) also observed that fear of failure in STEM students could lead to adaptive engagement through exploratory strategies. Adefisayo (2024) further supported this by showing a positive association between fear of failure and academic curiosity. However, Kashdan and Steger (2007) described curiosity as a coping response to stress, in which individuals seek to reduce uncertainty through information-seeking. In the present study, the observed association between curiosity and CPS was moderate, suggesting that curiosity may contribute to CPS, though its role is partial. While curiosity showed a significant mediation, other factors—such as grit—contributed independently. These results highlighted the multifaceted nature of CPS and suggest that future research could examine how curiosity interacts with other traits to influence academic outcomes.

### Indirect association of FoAF on CPS through Grit

The results strongly support the third study hypothesis, with a significant indirect association from FoAF to CPS via grit. This result underscores the role of grit in explaining how FoAF relates to CPS among undergraduate students. Specifically, students who experience FoAF tend to develop grit, which enhances their ability to solve problems creatively in academic settings. The significant indirect association of FoAF with CPS via grit is well supported by prior empirical research. Kim and Jang (2022) found that grit significantly enhances problem-solving ability among students, especially when they face distress or fear of academic failure, making grit a critical factor in overcoming challenges in learning environments. This has been echoed by Zhao et al. (2023), who highlighted grit as a central mechanism through which academic self-efficacy and perseverance manifest, particularly in stressful academic situations. Among undergraduate students, FoAF likely fosters grit to cope with academic stress. FoAF may lead students to engage more deeply with their academic work, not only out of curiosity but with a determined focus to overcome challenges. The indirect association of FoAF on CPS through grit reflects how grit enables students to maintain their effort and motivation over time, regardless of obstacles, thus facilitating CPS. Moreover, Wang et al. (2025) also support this, suggesting that grit, when combined with goal clarity and motivation, predicts students' ability to navigate ill-structured academic problems. Their findings indicate that students with higher grit are more likely to persevere in solving complex problems, which is essential for CPS in the face of failure or setbacks. Thus, the grit pathway in this study further emphasizes its central role in CPS among undergraduates dealing with FoAF.

The self-regulation theory and the achievement goal theory can explain the theoretical basis for this relationship. Zimmerman (2002) argued that self-regulation, which encompasses traits such as grit, is critical in helping students manage their emotional responses to academic challenges and maintain effort in the face of failure. Self-regulated students use grit as a mechanism to persist, even when faced with difficulties. In the case of FoAF, grit helps students overcome the anxiety associated with failure. It allows them to engage in long-term problem-solving efforts, which is essential for CPS. Furthermore, achievement goal theory (Ames, 1992) posits that students who adopt mastery goals, driven by a desire to improve and succeed, are more likely to exhibit grit and perseverance in the face of setbacks. This theory suggests that FoAF might shift students' focus toward performance-avoidant goals. Still, those with high grit can better reframe their fear as a motivator for sustained effort, thereby enhancing their problem-solving abilities. Duckworth and Quinn (2009) also highlighted that grit contributes to academic success by fostering long-term focus, which is essential in problem-solving tasks requiring significant effort and sustained attention over time. Thus, the indirect path through grit is grounded in the idea that FoAF does not necessarily hinder students but compels them to persevere and engage in more CPS strategies to overcome academic challenges.

The stronger indirect association of grit observed in this study suggested that grit accounted for a greater share of the indirect association between FoAF and CPS than curiosity did. While both traits explained variance in CPS, grit showed a stronger association, suggesting that perseverance-related traits may be more strongly associated with problem-solving outcomes in this sample. Existing literature supports the potential role of grit in academic persistence. For instance, Vallerand et al. (2003) emphasized that individuals high in perseverance are more likely to sustain effort in the face of obstacles and may be more inclined to apply creative strategies when faced with challenges. In the context of this study, grit was positively associated with both FoAF and CPS, suggesting it may be one pathway through which students channel academic fear into productive engagement. However, these findings reflect statistical relationships rather than evidence of psychological processes or causal mechanisms. Future longitudinal or experimental research is needed to explore how grit and curiosity jointly relate to CPS over time.

## Curvilinear sensitivity check and moderation

To examine whether the FoAF-to-CPS association departs from linearity at very high FoAF, CPS was compared across FoAF quartiles, and polynomial contrasts were tested; a regression including a centered quadratic FoAF term was also estimated. The pattern was approximately linear over the observed range: the linear trend was significant, the quadratic and cubic contrasts were not, and adding a quadratic term did not improve model fit. This exploratory evidence does not indicate a reliable downturn in CPS at the highest FoAF levels in this sample. Moreover, curiosity and grit, although trait-like, were specified as partial mediators to summarize concurrent co-variation among FoAF and CPS within a cross-sectional framework. Both traits index motivational engagement (curiosity) and persistence (grit), which are commonly associated with task focus and self-regulatory effort in academic contexts; each also shows reliable associations with CPS. Modeling them as mediators does not imply temporal precedence or causation; rather, it parsimoniously represents the FoAF–CPS association as partly accounted for by variance shared with these motivational traits, measured simultaneously. Exploratory moderation terms (FoAF × curiosity; FoAF × grit), estimated on mean-centered observed composites with age and gender controlled, did not reach significance, indicating that the FoAF–CPS association did not vary reliably across levels of curiosity or grit in this sample. In summary, the pattern was consistent with additive, correlational associations among concurrently measured variables, with curiosity and grit functioning as statistical bridges rather than altering the strength of the FoAF–CPS association.

### Study implications

The findings of this study offer several implications for academic theory and educational practice, particularly concerning students navigating FoAF. Theoretically, the results align with motivational and achievement goal theories by identifying curiosity and grit as traits associated with CPS. Although both traits mediated the FoAF–CPS relationship, grit exhibited a more substantial indirect association, suggesting a more persistence-oriented pathway. While the study did not test dynamic interactions or causality, the observed associations provide a basis for further theoretical exploration into how emotional and motivational constructs co-occur in academic settings. These findings support the importance of considering curiosity and grit in future research on student motivation and learning outcomes, especially under stress-inducing conditions.

In practical terms, the findings suggest that motivational traits such as grit and curiosity may be meaningful focal points in educational efforts to support students' CPS, especially in contexts where FoAF is prevalent. Although the current study does not test interventions, the observed associations indicate that students experiencing FoAF are not necessarily disengaged; instead, some may channel this emotion into persistence and adaptive engagement. This highlights the need for classroom environments that recognize fear as a common academic experience and provide scaffolding to help students manage it productively. In particular, initiatives that build perseverance—such as structured goal-setting programs, resilience training workshops, and progress-tracking systems—may align with the grit component highlighted in the model. Likewise, fostering curiosity can be supported through inquiry-based learning, problem-based learning, and exploratory classroom tasks that encourage students to seek out new knowledge. Educators might also consider blending structure and autonomy to balance emotional regulation with intellectual stimulation. These strategies, many of which have been tested in educational contexts, may not eliminate fear but can help reframe it as a challenge to be managed rather than a threat to avoid. Further research should test whether such approaches, especially when combined with emotional support, enhance students' ability to persist, explore, and solve problems in the face of academic stress.

### Limitations

Despite the valuable insights this study provides, several limitations must be considered when interpreting its findings. First, the use of convenience sampling limits the generalizability of the results. The sample was drawn from three universities in Putian City, China, which may not represent undergraduate students from other regions or cultural contexts. Given the influence of cultural norms on emotional responses and motivation, the observed relationships between FoAF, curiosity, grit, and CPS may not generalize beyond this demographic. Additionally, international students were omitted, limiting the study's applicability to more diverse educational populations. Second, the study relies solely on self-report measures, susceptible to common method bias. While we conducted Harman's single-factor test and found no substantial threat, it is still possible that social desirability, introspective limitations, or inaccurate self-assessment influenced participants' responses. The lack of behavioral or observational measures may also restrict the objectivity of the findings. Future studies may benefit from multi-method approaches, including performance-based or peer-reported assessments, to strengthen construct validity. Third, the study employed a cross-sectional design, restricting the ability to draw causal inferences. While significant associations were found, we cannot confirm their directionality. For example, curiosity or grit might relate to FoAF, rather than result from it. Longitudinal or experimental research is needed to examine the potential reciprocal or developmental relationships among these variables over time. Fourth, the study did not account for other potentially influential psychological variables—such as academic self-efficacy, coping strategies, or social support—which may confound or mediate the observed associations. Future studies should include such variables to build a more comprehensive model of academic problem-solving. Fifth, while the survey refers to motivation-related theories such as SDT and self-regulation theory, it does not directly examine goal orientations (e.g., mastery vs. performance), which are central to many motivational models. Future research could incorporate these constructs to further align with the theory. Lastly, while we reported internal consistency and path coefficients, we acknowledge the absence of direct comparisons with similar empirical studies. Benchmarking standardized associations or mediation strength against prior research would have enabled deeper contextual interpretation. This limitation constrains the broader theoretical positioning of our findings within the motivation and educational psychology literature. Finally, although age and gender were statistically controlled, the study did not conduct subgroup or stratified analyses by demographic variables such as gender, discipline, or academic year. Future research using multi-group SEM or stratified approaches could provide deeper insights into whether the observed relationships vary across student subpopulations.

## Conclusion

This study provides valuable insights into the complex relationships between FoAF, curiosity, grit, and CPS among undergraduate students. The findings underscore the significant mediating roles of curiosity and grit, with grit emerging as the stronger pathway linking FoAF to CPS. While curiosity motivates initial engagement and exploration, grit, through perseverance and sustained effort, plays a dominant role in enhancing CPS, especially under academic stress. These findings highlight that FoAF, often regarded as detrimental, can also catalyze adaptive motivational traits that support academic resilience and problem-solving. Beyond empirical results, this study offers critical theoretical contributions. Integrating self-regulation, self-determination, and achievement goal theories demonstrates how emotional experiences, such as fear, interact with motivational traits to shape academic outcomes. Methodologically, the study advances understanding by testing a parallel mediation model, clarifying the distinct yet complementary roles of curiosity and grit. The results also carry practical implications for teachers, educators, and policymakers. Interventions that nurture grit (e.g., goal-setting, persistence training, scaffolding challenges) and curiosity (e.g., inquiry-based learning, exploratory tasks) may help students reframe fear as a challenge rather than a barrier. For policymakers, embedding such traits into curricula and teacher-training programs could strengthen students' resilience and problem-solving capacity in increasingly demanding academic contexts. Finally, this study opens pathways for future research. Longitudinal designs are needed to examine causal and developmental dynamics between FoAF, curiosity, grit, and CPS over time. Comparative studies across different educational levels (e.g., high school vs. postgraduate) and cross-country investigations would also help determine the generalizability of these relationships across cultural and institutional contexts. By pursuing these directions, future scholarship can extend the present findings and offer more nuanced insights into how motivational traits mediate the impact of academic fears on learning outcomes.

## Data Availability

The raw data supporting the conclusions of this article will be made available by the authors, without undue reservation.
